# Effects of Novel Isoform-Selective Phosphoinositide 3-Kinase Inhibitors on Natural Killer Cell Function

**DOI:** 10.1371/journal.pone.0099486

**Published:** 2014-06-10

**Authors:** Sung Su Yea, Lomon So, Sharmila Mallya, Jongdae Lee, Kamalakannan Rajasekaran, Subramaniam Malarkannan, David A. Fruman

**Affiliations:** 1 Department of Molecular Biology & Biochemistry, and Institute for Immunology, University of California Irvine, Irvine, California, United States of America; 2 Department of Biochemistry, College of Medicine, Inje University, Busan, Korea; 3 Laboratory of Molecular Immunology and Immunotherapy, Blood Research Institute, Milwaukee, Wisconsin, United States of America; University of Torino, Italy

## Abstract

Phosphoinositide 3-kinases (PI3Ks) are promising targets for therapeutic development in cancer. The class I PI3K isoform p110α has received considerable attention in oncology because the gene encoding p110α (*PIK3CA*) is frequently mutated in human cancer. However, little is known about the function of p110α in lymphocyte populations that modulate tumorigenesis. We used recently developed investigational inhibitors to compare the function of p110α and other isoforms in natural killer (NK) cells, a key cell type for immunosurveillance and tumor immunotherapy. Inhibitors of all class I isoforms (pan-PI3K) significantly impaired NK cell-mediated cytotoxicity and antibody-dependent cellular cytotoxicity against tumor cells, whereas p110α-selective inhibitors had no effect. In NK cells stimulated through NKG2D, p110α inhibition modestly reduced PI3K signaling output as measured by AKT phosphorylation. Production of IFN-γ and NK cell-derived chemokines was blocked by a pan-PI3K inhibitor and partially reduced by a p110δinhibitor, with lesser effects of p110α inhibitors. Oral administration of mice with MLN1117, a p110α inhibitor in oncology clinical trials, had negligible effects on NK subset maturation or terminal subset commitment. Collectively, these results support the targeting of *PIK3CA* mutant tumors with selective p110α inhibitors to preserve NK cell function.

## Introduction

The immune system plays both negative and positive roles in cancer development [1]. Lymphocyte subsets including NK cells and cytotoxic T lymphocytes can recognize and kill tumor cells. Conversely, inflammatory cells can promote tumor initiation and development, and regulatory T cells maintain an immunosuppressive milieu in tumors and draining lymph nodes. Drugs developed against molecular targets in tumors have the potential to modify the function of all of these leukocyte populations, enhancing or interfering with immunotherapeutic strategies [2], [3]. Therefore, it is critical to define the effects of emerging cancer therapies on immune function.

A major target of experimental cancer drugs is the PI3K signaling pathway, which is aberrantly activated in most human tumors [4]–[6]. In recent years, candidate agents with good pharmacological properties and acceptable toxicity in animals have entered clinical trials for oncology. There are two main classes of PI3K inhibitor. The first class includes compounds selective for individual class I PI3K isoforms (p110α, p110β, p110γ or p110δ). The other class encompasses “pan-PI3K” inhibitors with similar potency against all class I PI3K enzymes. Isoform-selective inhibitors targeting either p110α or p110δ have received particular attention in oncology [4]–[6]. The rationale for p110α-selective inhibitors is that activating mutations in *PIK3CA*, encoding p110α, are common in epithelial tumors [7], [8]. Preclinical studies indicate that p110α-selective compounds are equally effective as pan-PI3K inhibitors at reducing growth of *PIK3CA* mutant tumor cells [9]–[11]. The main factor driving interest in p110δhas been the dramatic and unpredicted success of p110δinhibitors in early clinical trials of B cell malignancies [4], [12]. Compounds with activity against p110β or p110γ might also suppress growth of certain cancers [13], [14].

Recent advances in medicinal chemistry have produced refined chemical tools to probe the function of individual PI3Ks in different cell types [4], [6]. In this study we compared pan-PI3K and isoform-selective inhibitors in assays of NK cell function. NK cells are important for host defense to viral infections, killing virally-infected cells directly and producing cytokines that influence other cells of innate and adaptive immunity [15], [16]. NK cells are also critical for tumor immunosurveillance and can be used in adoptive immunotherapy [17]. NK cells display natural cell-mediated cytotoxicity (CMC) against cancer cells through the detection of stress ligands (also known as “induced self”), mediated by NKG2D and other activating receptors, or through recognition of “missing self” when tumor cells have low surface expression of MHC class I molecules. In addition, NK cells mediate antibody-dependent cellular cytotoxicity (ADCC) through Fcγreceptor-dependent recognition of antibody-coated targets. There is evidence that ADCC mediated by NK cells and monocytes plays a major role in destruction of tumor cells in humans treated with therapeutic antibodies such as cetuximab, trastuzumab and rituximab [18], [19]. Ideally, targeted anti-cancer agents should not interfere with the ability of NK cells to produce cytokines or kill tumor cells. However, various NK receptors activate PI3K and broad spectrum PI3K inhibitors strongly suppress NK cell function [20]–[24]. Until recently it was not possible to test the role of p110α in NK cells; mutations in the mouse p110α gene are embryonic lethal [25] and selective inhibitors were not available. Using newly developed compounds with high selectivity for p110α [11], we tested the hypothesis that p110α inhibitors have lesser effects than pan-PI3K inhibitors on crucial functions of NK cells. The results support this prediction and show that multiple PI3K isoforms have overlapping and largely redundant roles.

## Results

### Pan-PI3K inhibitors strongly suppress NK CMC

**Figure 1:**
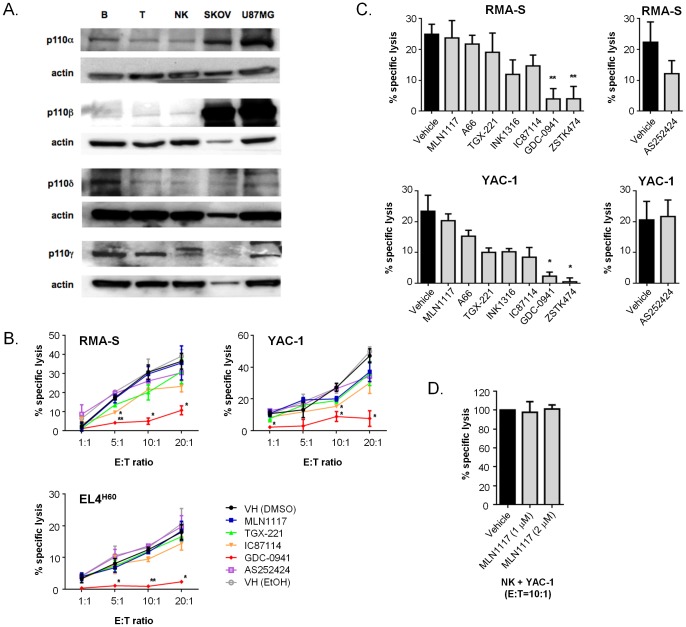
NK cells express all class I PI3K isoforms but isoform-selective inhibitors have little effect on cytotoxicity. (**A**) Immunoblots were prepared using equivalent protein amounts from lysates of purified NK cells, T cells, B cells or two solid tumor cell lines. Blots were probed with PI3K isoform-specific antibodies and for β-actin as a loading control. (**B**) RMA-S, YAC-1, and EL4^H60^ cells were labeled with calcein AM and co-cultured with NK cells at the indicated E:T ratios in the presence of 1 µM indicated inhibitors (TGX-221 was 0.5 µM) for 2 h. (**C**) RMA-S and YAC-1 cells were labeled with calcein AM and co-cultured with NK cells at 10∶1 E:T ratio in the presence of 1 µM indicated inhibitors for 2 h. (**D**) NK cells were treated with a higher concentration of MLN1117 (2 µM) and incubated with calcein AM-labeled YAC-1 cells at 10∶1 E:T ratio. Percentage specific lysis was calculated from pentaplicate cultures as follows: ((mean experimental release - mean spontaneous release)/(mean maximum release -mean spontaneous release)) x 100. The values are presented as the means ± SEM from three independent experiments. *p<0.05 and **p<0.01, as determined by the unpaired Student's *t* test using Sigma Plot 10 software and as compared to the vehicle control group.

Several studies have shown that PI3K inhibitors suppress NK cell-mediated cytotoxicity towards tumor cell lines. Early reports employed non-selective compounds such as wortmannin and LY294002 that also inhibit other cellular enzymes at the concentrations used [20], [24]. Additional evidence that PI3K is required for NK CMC has emerged from genetic and pharmacological inhibition of the PI3K isoforms p110γ or p110δ [24], [26]–[30]. To confirm the PI3K-dependence of NK cell functions under our experimental conditions, we used the selective pan-class I inhibitors ZSTK474 [31], [32] and GDC-0941 [33]–[35]. As shown in **Table 1**, both compounds inhibit all four class I PI3K enzymes in the low to mid-nanomolar range *in vitro*; in cellular assays these compounds block PI3K signaling selectively when used at 0.1–1 µM. For cytotoxicity assays, we first used purified murine NK cells (**Fig. S1**) expanded and activated for one week with recombinant human IL-2. These cells effectively lysed lymphoma target cells lacking MHC class I (RMA-S) or cells expressing the H60 stress ligand for NKG2D (EL4^H60^), compared to their respective control cell lines RMA and EL4 (**Fig. S2A**). ZSTK474 strongly suppressed CMC against both target cells over a wide range of E:T ratios (**Fig. S2A**). The effect of ZSTK474 was dose-dependent, with maximal efficacy at 1 µM (**Fig. S2B**). GDC-0941 also suppressed CMC towards RMA-S and EL4^H60^ cells (**Fig. 1B, C**). Both ZSTK474 and GDC-0941 blocked NK cell killing of YAC-1 cells, a commonly used lymphoma target cell line naturally expressing stress ligands (**Fig. 1B, C**). These experiments establish conditions in which CMC is absolutely dependent on class I PI3K activity.

**Table 1:** Selectivity and effective concentrations of inhibitors used in this study (reported in the following references: 11, 26, 31, 34, 38, 40).

Inhibitor	Selectivity	Effective Concentrations
MLN1117	p110α	*In vitro* IC_50_: 15 nM (p110α), >1 µM (other isoforms) Cellular Assays: selective for p110α at 1 µM
A66	p110α	IC_50_: 32 nM (p110α), >1 µM (other isoforms) Cellular Assays: 1 µM
TGX-221	p110β	IC_50_: 5 nM (p110β), 100 nM (p110δ), >5 µM (other isoforms) Cellular Assays: 500 nM
INK1316	Dual p110α/β	IC_50_: 10 nM (p110α), 8 nM (p110β), >750 nM (other isoforms) Cellular Assays: 1 µM
AS252424	p110γ	IC_50_: 30 nM (p110γ), >1 µM (other isoforms) Cellular Assays: 1 µM
IC87114	p110δ	IC_50_: 70 nM (p110α), >1 µM (other isoforms) Cellular Assays: 1 µM
GDC-0941	Pan-class I PI3K	IC_50_: 3 nM (p110α), 33 nM (p110β), 75 nM (p110γ), 3 nM (p110δ) Cellular Assays: 250 nM–1 µM
ZSTK474	Pan-class I PI3K	IC_50_: 16 nM (p110α), 44 nM (p110β), 49 nM (p110γ), 4.6 nM (p110δ) Cellular Assays: 250 nM–1 µM

### Isoform-selective PI3K inhibitors have minimal effect on NK cytotoxicity

Murine NK cells express each of the four class I PI3K isoforms as detected by immunoblot (**Fig. 1A**). The expression levels in NK cells were similar to T lymphocytes, both of which expressed much less p110α and p110β than solid tumor cell lines SKOV3 and U87MG (**Fig. 1A**). Expression of p110α in NK cells and T cells was slightly lower than in B cells (**Fig. 1A**), a cell type in which p110α inhibition significantly reduces PI3K signaling output following antigen receptor crosslinking [11].

To define the contributions of individual PI3K isoforms to the overall CMC response in NK cells, we employed a panel of inhibitors we described recently in a study of T and B lymphocytes [11]. MLN1117 (formerly INK1117) and A66 are structurally distinct compounds selective for p110α *in vitro* (**Table 1**) and are selective for p110α in cells when used at 1 µM [11], [36]. TGX-221 is a well-characterized p110β-selective inhibitor when used at 500 nM (**Table 1**) [37], [38]. We also tested the compound INK1316, which inhibits both p110α and p110β*in vitro* (**Table 1**) [11]. To inhibit p110δ we used IC87114, a highly selective compound (**Table 1**) that has been widely used in lymphocyte studies [39], [40] and impairs NK cell chemotaxis at 1 µM [28]. At concentrations higher than 1 µM, IC87114 has off-target effects [40]. To inhibit p110γ we used the compound AS252424 (**Table 1**) at a concentration (1 µM) used in previous studies of NK cells [24], [28]. None of the compounds were directly toxic to either the NK cells or the target cells (**Fig. S3**).

Selective inhibition of p110α, p110β or p110γ did not significantly reduce NK cell-mediated killing of RMA-S, EL4^H60^ or YAC-1 cells (**Fig. 1B, C**). Raising the concentration of MLN1117 to 2 µM still did not inhibit killing of YAC-1 cells (**Fig. 1D**). The dual p110α/p110β inhibitor INK1316 likewise did not significantly reduce CMC towards RMA-S and YAC-1 cells (**Fig. 1C**). The p110δ inhibitor IC87114 modestly reduced killing of RMA-S and YAC-1 cells at some but not all E:T ratios (**Fig. 1B**), but the magnitude of the inhibition was much less than achieved with GDC-0941.

**Figure 2:**
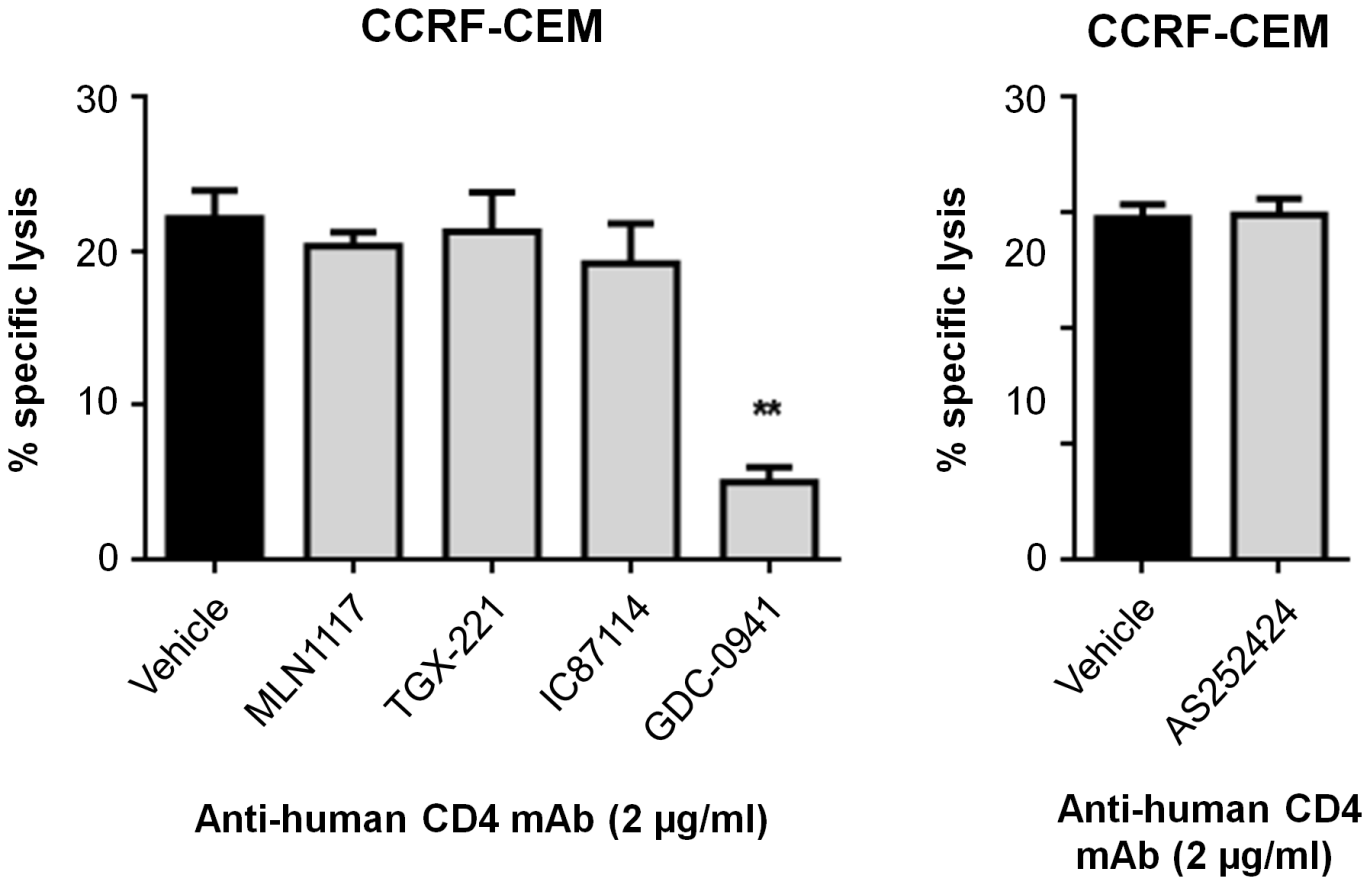
A pan-PI3K inhibitor, but not isoform-selective inhibitors, suppresses ADCC. CCRF-CEM, human acute T lymphocytic leukemia cell line was labeled with calcein AM, incubated with mouse anti-human CD4 mAb, and then co-cultured with NK cells at 10∶1 E:T ratio in the presence of indicated inhibitors for 2 h. Culture supernatants were collected and calcein fluorescence was measured and analyzed as in Figure 1. The values are presented as the means ± SEM from three independent experiments. *p<0.05 and **p<0.01, as determined by the unpaired Student's *t* test using Sigma Plot 10 software and as compared to the vehicle control group.

As a model of ADCC, we cultured mouse NK cells together with a human CD4^+^ T cell lymphoma line (CCRF-CEM) in the presence of mouse anti-human CD4 monoclonal antibody. Similar to the CMC results, ADCC was strongly reduced by the pan-PI3K inhibitor GDC-0941 but not by isoform-selective inhibitors targeting p110α, p110β, p110γ or p110δ (**Fig. 2**). Together these experiments establish that individual class I PI3K isoforms have overlapping, redundant roles in NK cell-mediated killing of target cells via recognition of missing self (RMA-S), stress ligands (YAC-1 and EL4-H60) or antibody (CCRF-CEM plus anti-CD4).

**Figure 3:**
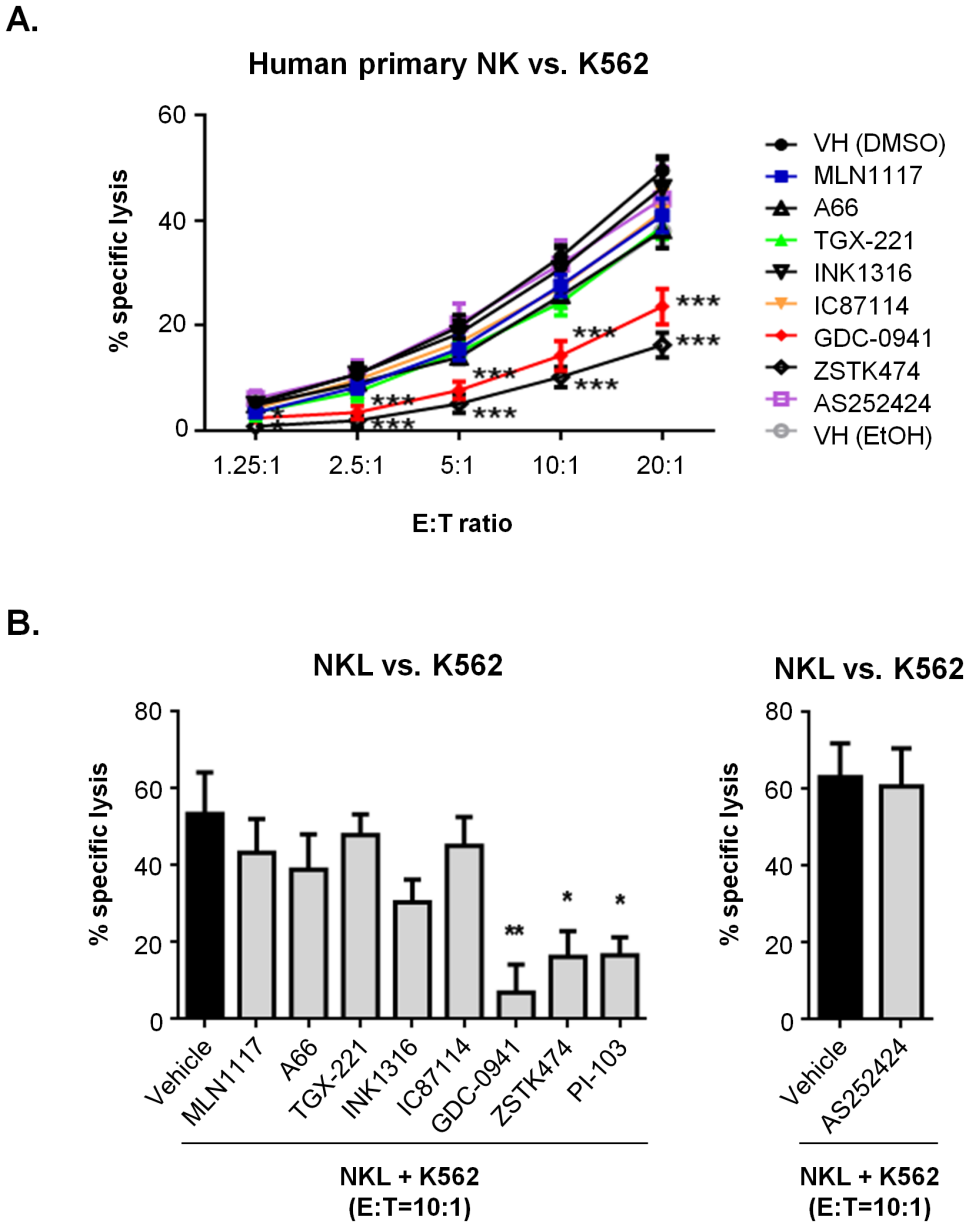
Isoform-selective inhibitors have little effect on cytotoxicity of human NK cells. (**A**) K562 cells were labeled with ^51^Cr and co-cultured with human primary NK cells at the indicated E:T ratios in the presence of 1 µM indicated inhibitors (TGX-221, GDC-0941, and ZSTK474 were 0.5 µM) for 2 h. Specific ^51^Cr release was measured as described [46]. (**B**) K562 cells were labeled with calcein AM and co-cultured with human NKL cells at 10∶1 E:T ratio in the presence of 1 µM indicated inhibitors (TGX-221 was 0.5 µM) for 2 h. Culture supernatants were collected and calcein fluorescence was measured and analyzed as in Figure 1. The data are expressed as the means ± SEM of three independent experiments. An asterisk denotes any response that is significantly different from the vehicle control group as determined by the unpaired Student's *t* test using Sigma Plot 10 software (*, *p*<0.05, **, *p*<0.01, and ***, *p*<0.001).

To extend these findings to human NK cells, we assessed killing of K562 target cells by primary human NK cells from three different donors. As in mouse NK cells, the pan-PI3K inhibitors GDC-0941 and ZSTK474 significantly suppressed CMC at all E:T ratios (**Fig. 3A**) confirming the PI3K-dependence of the response. In this system, we observed again that isoform-selective inhibitors had minimal effect. Similar results were obtained using the human NK cell lines NKL (**Fig. 3B**) and NK92 (**Fig. S4**).

### Isoform-selective PI3K inhibitors partially reduce AKT phosphorylation in stimulated NK cells

**Figure 4:**
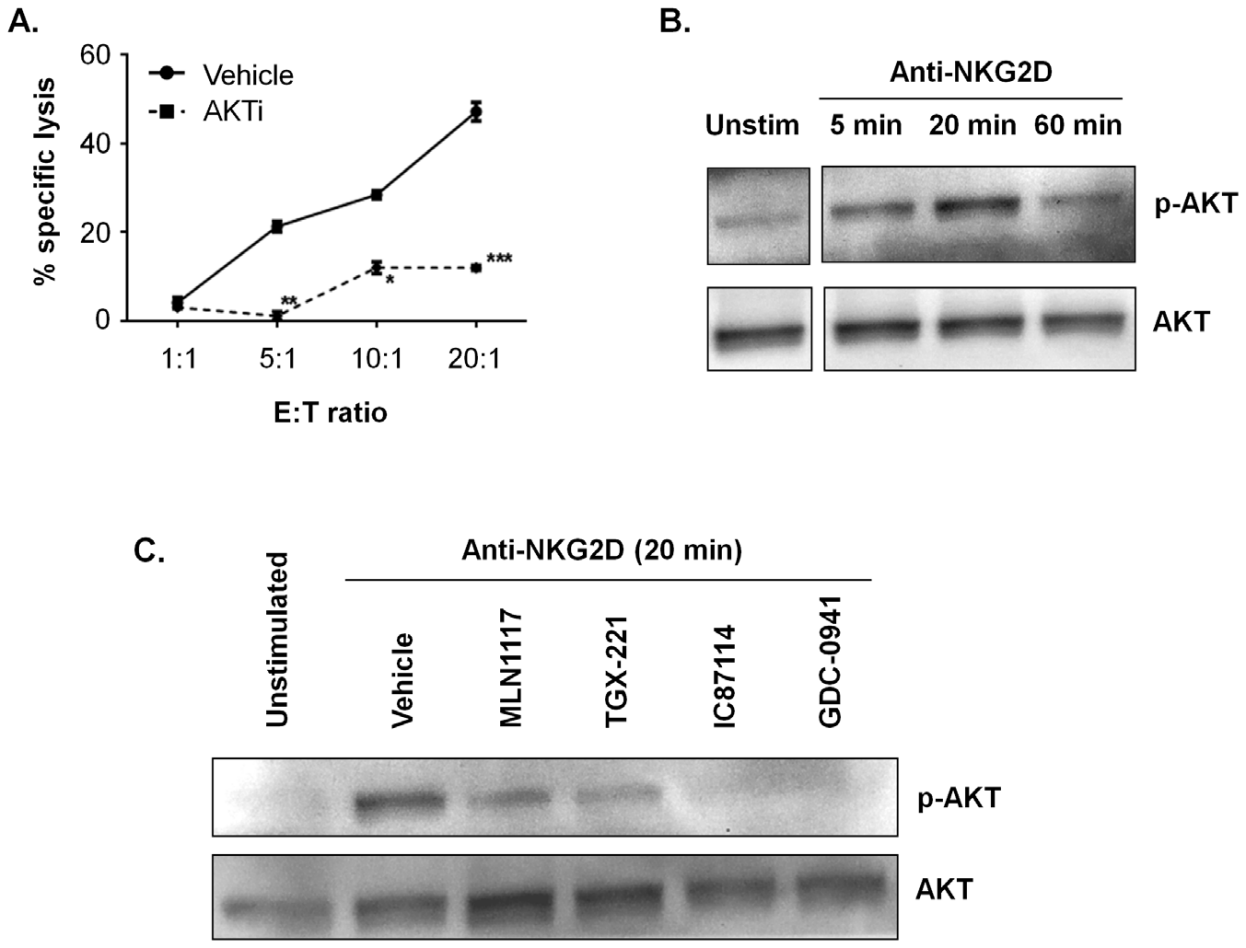
Isoform-selective inhibitors partially reduce AKT phosphorylation in anti-NKG2D-stimulated NK cells. NK cells from C57BL/6 mice were purified from spleen and expanded for 7–8 days in IL-2. (**A**) YAC-1 cells were labeled with calcein AM and co-cultured with NK cells at the indicated E:T ratios in the presence of AKTi, a specific inhibitor of AKT1 and AKT2, for 2 h. Culture supernatants were collected and calcein fluorescence was measured. Percentage specific lysis was calculated from pentaplicate cultures as follows: ((mean experimental release - mean spontaneous release)/(mean maximum release - mean spontaneous release)) x 100. Data presented are means ± SEM from three independent experiments. An asterisk denotes any response that is significantly different from the vehicle control group as determined by the unpaired Student's *t* test using Sigma Plot 10 software (*, *p*<0.05, **, *p*<0.01, and ***, *p*<0.001). (**B**) NK cells were stimulated with plate-bound anti-NKG2D mAb for the indicated times. Whole-cell lysates were probed for pAKT (S473) and reprobed for total AKT. (**C**) NK cells were pretreated with 1 µM of the indicated inhibitors (TGX-221 was 0.5 µM) before anti-NKG2D stimulation for 20 minutes. Similar results were obtained in two additional experiments, though p110δ inhibitor IC87114 caused less reduction in pAKT than GDC-0941 in other experiments.

To determine the impact of different PI3K inhibitors on a proximal signaling event, we measured phosphorylation of AKT. Membrane recruitment, phosphorylation and activation of AKT is a critical PI3K-dependent signal in many cell types. In accord, a selective AKT inhibitor strongly suppressed NK cell cytotoxicity towards YAC-1 cells (**Fig. 4A**). In NK cells stimulated with anti-NKG2D, AKT phosphorylation increased within 5 minutes and reached a maximum around 20 minutes (**Fig. 4B**). Pretreatment with GDC-0941 abolished the increase in pAKT (**Fig. 4C**), showing that the measured phosphorylation is dependent on class I PI3Ks. Selective inhibitors of p110α, p110β or p110δ partially reduced pAKT (**Fig. 4C**). These results suggest that each isoform contributes to PI3K signaling output following NK stimulation through NKG2D.

### Redundant roles of different PI3K isoforms in NK cell production of cytokines and chemokines

**Figure 5:**
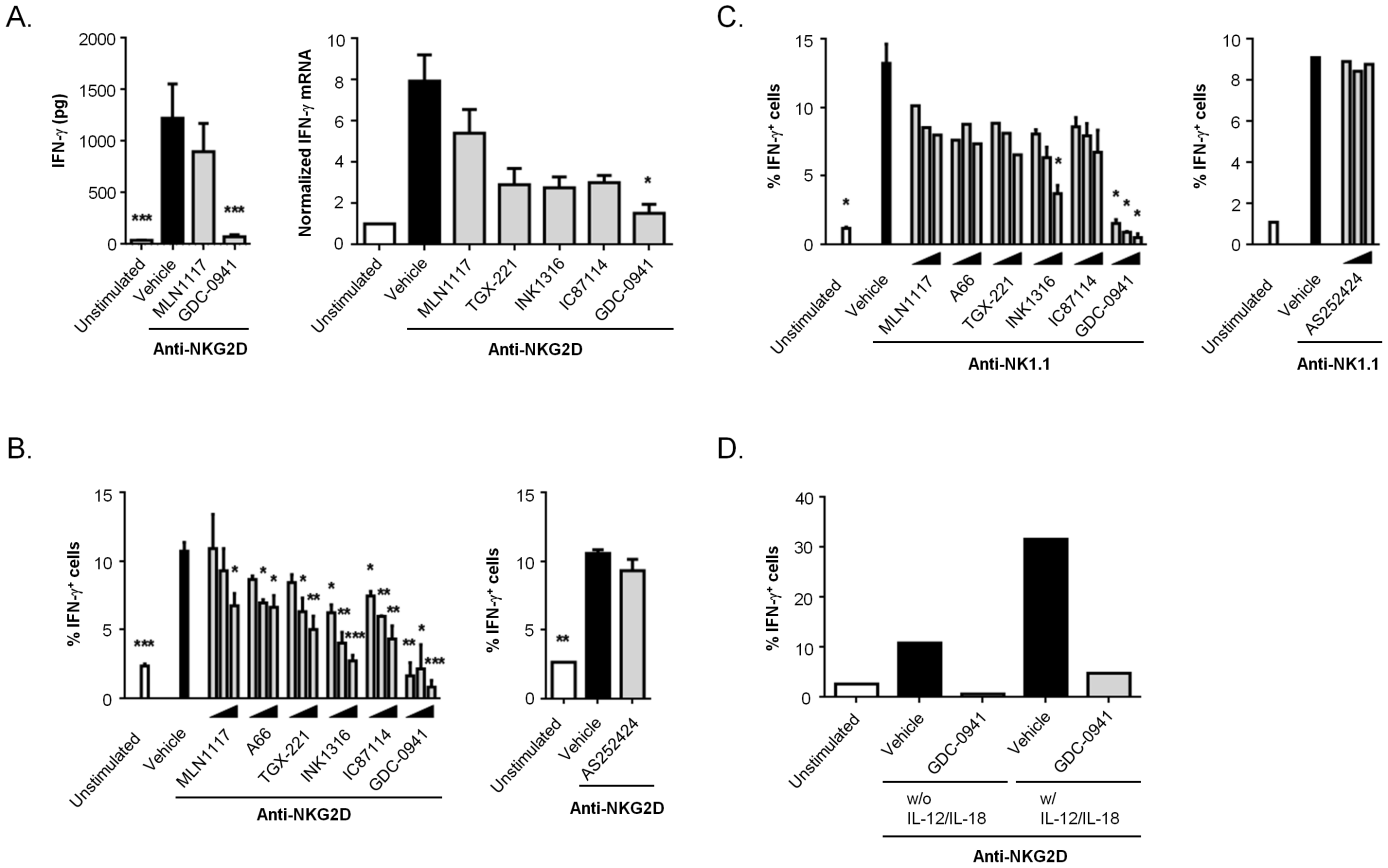
Selective p110α inhibitors have lesser effect on IFN-γ production in anti-NKG2D- or anti-NK1.1-stimulated NK cells. IL-2-expanded mouse NK cells were stimulated with plate-bound anti-NKG2D mAb (**A–B**) or anti-NK1.1 mAb (C) in the presence of 1 µM indicated inhibitors (TGX-221 was 0.5 µM) (A) or 250 nM, 500 nM, and 1 µM indicated inhibitors (B–C). After 18 h stimulation, the supernatants were collected and IFN-γ secretion was determined by ELISA (A, left). After 6 h stimulation, total RNA was isolated and IFN-γ mRNA expression was determined by real-time RT-PCR using SYBR Green (A, right). The relative transcript quantities were calculated as described in Materials and Methods section and normalized using the unstimulated group defined as 1. After 18 h stimulation, the NK cells were harvested and the intracellular IFN-γ level was determined by flow cytometry (B–C). Brefeldin A was added for the last 4 h before cell harvest and IFN-γ production was measured in CD3^−^NK1.1^+^ NK cells by intracellular staining. The data are expressed as the means ± SEM of three independent experiments. Statistical analysis was performed with one-way ANOVA using Prism 6 (GraphPad Software, Inc.) to compare the differences between vehicle and each inhibitor-treated group. *, *p*<0.05, **, *p*<0.01, and ***, *p*<0.001. (**D**) NK cells were pre-treated with vehicle (0.1% DMSO) or 1 µM GDC-0941 for 15 min, followed by stimulation with plate-bound anti-NKG2D mAb in the absence or presence of IL-12 and IL-18 for 18 h. The intracellular IFN-γ level was determined as in B–C.

NK cells produce many different cytokines and chemokines upon engagement of activating receptors [16]. These secreted factors influence several cell types of the innate and adaptive immune systems. IFN-γ secretion by NK cells is particularly important for controlling replication of intracellular pathogens and promoting T helper type I responses [41]. We measured IFN-γ production following stimulation with plate-bound antibodies to activating receptors (NKG2D or NK1.1), after a 15 min pretreatment with drugs. In cells stimulated with anti-NKG2D for 18 hours, GDC-0941 but not MLN1117 suppressed production of IFN-γ protein measured by ELISA (**Fig. 5A**, *left*). Anti-NKG2D strongly induced IFN-γ mRNA after 6 hours, and this response was nearly abolished by GDC-0941 (**Fig. 5A**, *right*). MLN1117 and other isoform-selective inhibitors did not significantly reduce IFN-γ mRNA at this time point, though there was a trend to reduced mRNA in cells treated with TGX-221, INK1316 or IC87114 (**Fig. 5A**, *right*). To determine the percentage of NK cells producing IFN-γ, we performed intracellular cytokine staining. 18 hours after stimulation with anti-NKG2D or anti-NK1.1, approximately 10–15% of NK1.1^+^ cells expressed IFN-γ at a level detectable in this assay (**Fig. 5B, C** and **Fig. S5**). GDC-0941 reduced the percentage of IFN-γ^+^ NK cells to background levels at all drug concentrations tested (**Fig. 5B, C**). The two p110α inhibitors modestly reduced the percentage of IFN-γ^+^ NK cells in response to anti-NKG2D, but this was not statistically significant when anti-NK1.1 was used (**Fig. 5B, C**). Isoform-selective inhibitors of p110β or p110δ also had a significant effect on the response to anti-NKG2D but not anti-NK1.1, yet none of these compounds approached the complete inhibition achieved by GDC-0941 (**Fig. 5B, C**).

**Figure 6:**
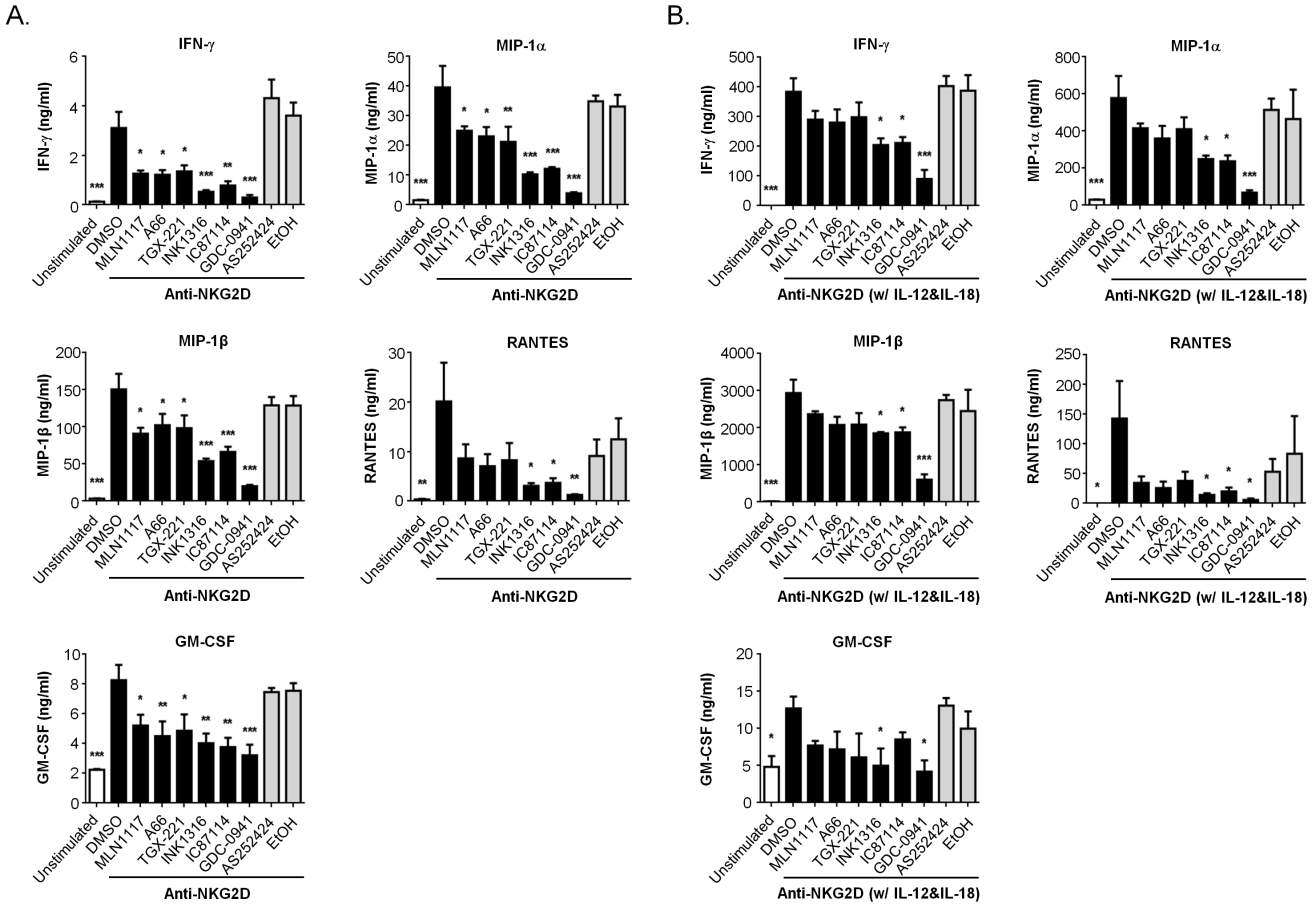
Selective p110α inhibitors have lesser effect on cytokine/chemokine production in NK cells. NK cells from C57BL/6 mice were purified from spleen and expanded for 7–8 days in IL-2. (A–B) The NK cells were pre-treated with vehicle (0.1% DMSO or 0.1% ethanol) or 1 µM indicated inhibitors (TGX-221 was 0.5 µM) for 15 min, followed by stimulation with plate-bound anti-NKG2D mAb in the absence (**A**) or presence (**B**) of IL-12 and IL-18 for 18 h. The supernatants were collected and analyzed by Multiplex assay. The data are expressed as the means ± SEM of four independent NK cell preparations that were stimulated and analyzed concurrently. Statistical analysis was performed with one-way ANOVA using Prism 6 (GraphPad Software, Inc.) to compare the differences between vehicle and each inhibitor-treated group. *, *p*<0.05, **, *p*<0.01, and ***, *p*<0.001.

Next we measured production of a large panel of cytokines and chemokines using a bead based bioplex assay. We stimulated cells from four different mice with anti-NKG2D either in the absence or presence of exogenous IL-12 and IL-18, which enhance cytokine production by NK cells (**Fig. 5D**). The amounts of classical NK-derived cytokines (IFN-γ, GM-CSF) and chemokines (MIP-1α, MIP-1β, RANTES; also known as CCL3, CCL4, CCL5) increased significantly in supernatants from anti-NKG2D-stimulated cells compared to unstimulated control (**Fig. 6A, B**). In the presence of IL-12 and IL-18, NK cells produced 70–200 fold more IFN-γ and chemokines but only slightly more GM-CSF (compare **Fig. 6B** to **6A**). The pan-PI3K inhibitor GDC-0941 strongly and significantly reduced production of each cytokine/chemokine under both stimulation conditions (**Fig. 6A, B**). The p110δ-selective inhibitor IC87114 caused an intermediate reduction of these cytokines and chemokines, which was statistically significant except for GM-CSF produced in the presence of IL-12 and IL-18 (**Fig. 6A, B**). A key observation was that in NK cells treated with the p110αinhibitors or p110β inhibitor, cytokine production was reduced to a lesser degree than in cells treated with IC87114. These reductions were not statistically significant in NK cells stimulated in the presence of IL-12 and IL-18.

**Figure 7:**
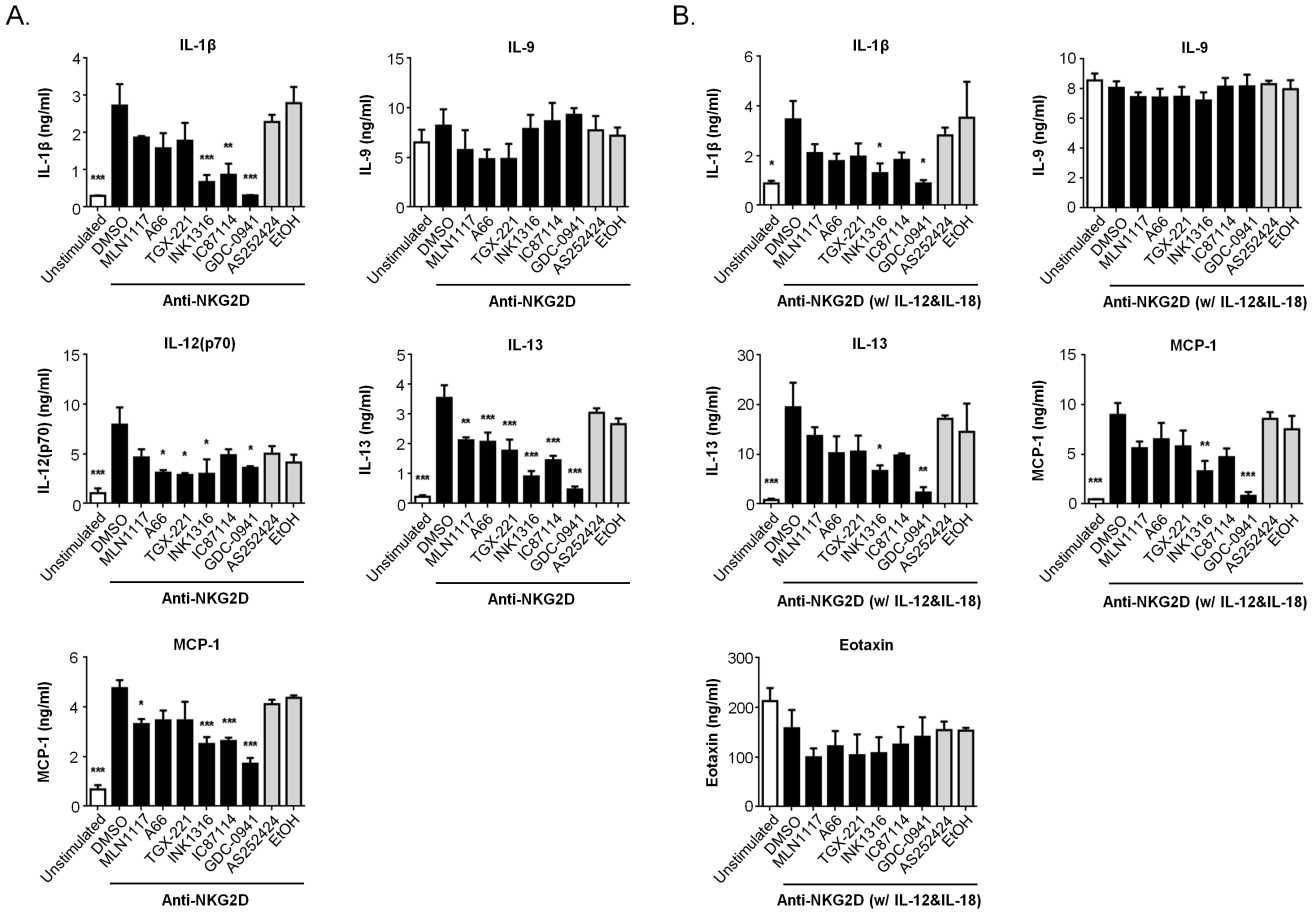
Effects of PI3K inhibitors on additional cytokines and chemokines produced by NK cells. Data from additional bioplex analytes are shown as in Figures 5 and 6. Displayed are analytes present at concentrations greater than 1 ng/ml in at least one condition, based on the Bioplex product information showing a limit of detection between 0.1–1 ng/ml.

Several other cytokines and chemokines were produced at lower levels than the classical NK cell products presented in Figure 6. **Figure 7** shows results for products present above 1 ng/ml in supernatants of stimulated cells. We chose this cut-off based on the sensitivity of the detection assay (see figure legend). IL-9 was produced constitutively (>5 ng/ml) and this was not strongly increased by stimulation with anti-NKG2D or IL-12/IL-18 (**Fig. 7A, B**). PI3K inhibitors including GDC-0941 did not reduce IL-9 production, indicating that the PI3K inhibitors were not generally toxic to NK cells or the secretory pathway. In NK cells cultured in the absence of IL-12/IL-18, anti-NKG2D increased production of IL-1β, IL-12 (p70), IL-13, and MCP-1 and all were reduced by GDC-0941 and the dual p110α/βinhibitor (**Fig. 7A**). IC87114 significantly reduced production of IL-1β, IL-13, and MCP-1 but not IL-12 (p70). The p110αinhibitors and p110β inhibitor had variable effects that were significant for some products. NK cells cultured in IL-12 and IL-18 produced a similar set of cytokines (IL-1β, IL-13, and MCP-1), but at higher amounts particularly for IL-13 (**Fig. 7B**). Again, GDC-0941 and INK1316 significantly reduced production of IL-1β, IL-13, and MCP-1. Under these conditions, inhibitors of individual PI3K isoforms caused a trend towards lower cytokine/chemokine production but these were not significant.

Considered together, the bioplex data combined with intracellular cytokine staining suggest that p110α, p110β and p110δ contribute to the overall PI3K signal driving cytokine production, with p110δ mediating the greatest quantitative input in some cases. However, each of the isoform-selective inhibitors has a lesser impact on cytokine production compared to pan-PI3K inhibition in these *in vitro* studies. This pattern correlates closely with the results from pAKT measurements in **Figure 4**, and with cytotoxicity results in **Figure 1**.

### Treatment with PI3K inhibitors in vivo has minimal effect on NK cell maturation or survival

**Figure 8:**
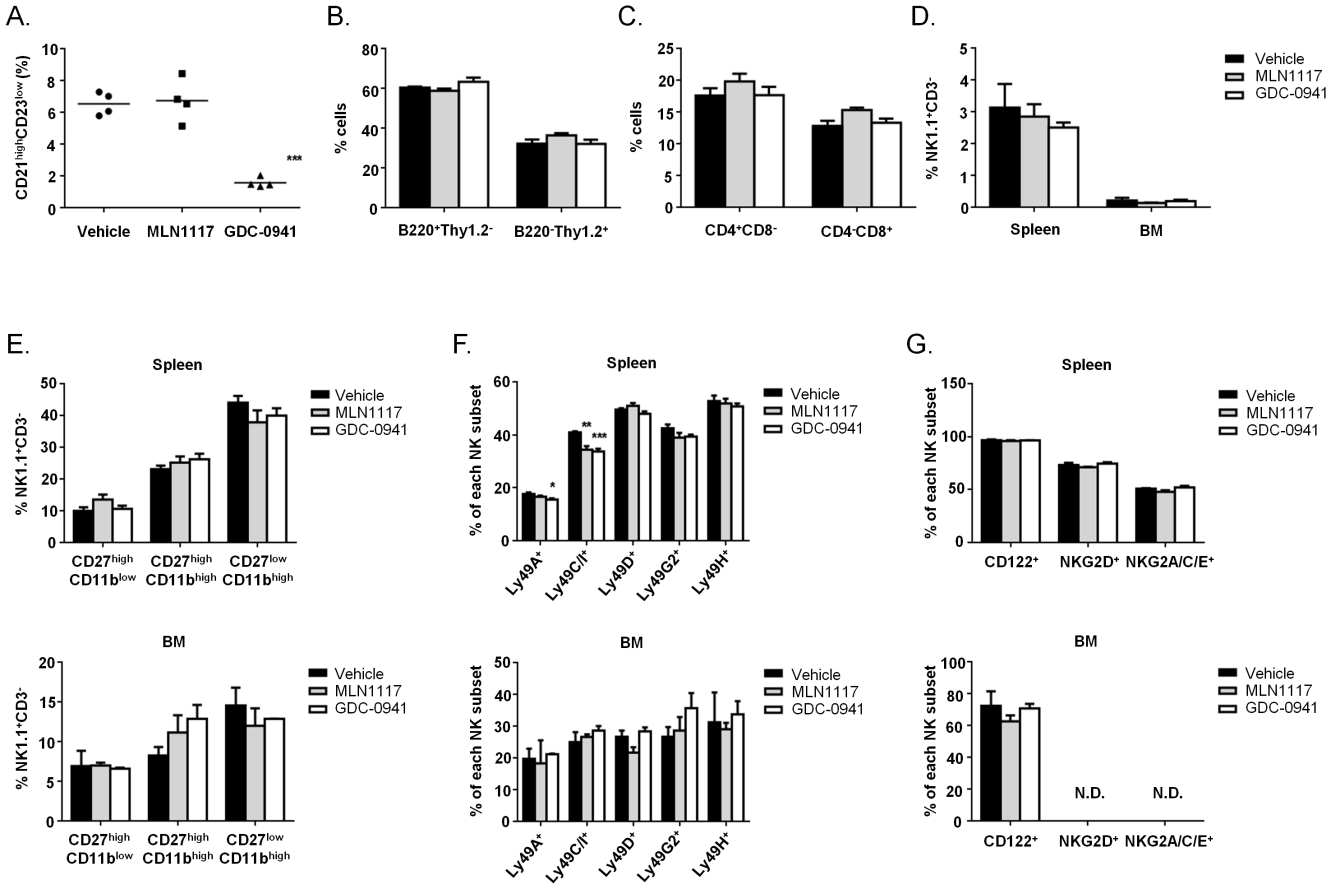
PI3K inhibitors have minimal effect on the development of NK cells *in vivo*. Wild-type C57BL/6 mice were orally dosed with vehicle, the p110α selective inhibitor MLN1117 (60 mg/kg), or the pan-PI3K inhibitor GDC-0941 (70 mg/kg) for 7 days (n = 4). Spleen and bone marrow (BM) were isolated and analyzed for NK cell development by flow cytometry. (**A**) Splenocytes were stained with CD21 and CD23 for marginal zone (MZ) B cell population. Among the B220^+^ population, CD21^high^CD23^low^ cells were gated for MZ B cell population. (**B, C**) Percentage of B/T cells and Th/Tc cells in spleen were determined using surface staining with anti-B220/anti-Thy1.2 mAbs and anti-CD4/anti-CD8 mAbs, respectively. (**D**) NK cells as specified by CD3^−^NK1.1^+^ were gated and analyzed in single-cell suspension of spleen and BM, respectively. (**E**) Spleen- or BM-derived CD3^−^NK1.1^+^ NK cells were analyzed for NK subset based on the expression level of CD11b and CD27. (**F**) Spleen- or BM-derived CD3^−^NCR1^+^ NK cells were analyzed for maturation marker, Ly49A, LY49C/I, Ly49D, Ly49G2, and Ly49H. (**G**) Spleen- or BM-derived CD3^−^NCR1^+^ NK cells were analyzed for the expression of CD122, NKG2D, and NKG2A/C/E. The data are expressed as the means ± SEM. Statistical analysis was performed with the unpaired Student's *t* test using Sigma Plot 10 software to compare the differences between vehicle and each inhibitor-treated group. *, *p*<0.05, **, *p*<0.01, and ***, *p*<0.001.

Previously we showed that mouse B cell subset frequencies are altered by *in vivo* dosing with the pan-PI3K inhibitor GDC-0941 but not by the p110α inhibitor MLN1117 [11]. To test the effect of these drugs on NK cell frequencies and maturation state, mice were given daily oral doses of GDC-0941 or MLN1117 for one week, or treated with vehicle control. We used doses and formulations shown previously to suppress growth of *PIK3CA* mutant tumors in xenograft models [10], [35]. As we reported [11], GDC-0941 but not MLN1117 significantly reduced the percentage of B cells with a marginal zone phenotype (CD21^hi^CD23^lo^) (**Fig. 8A**). Neither compound altered the percentages of T cells and B cells or the fractions of CD4 and CD8 T cells in spleen, or the percentages of NK cells in spleen and bone marrow (**Fig. 8B, C, D**).

In mice treated with GDC-0941 or MLN1117 for one week, there was no significant change in the percentages of NK cells representing different maturation stages based on staining for CD27 and CD11b (**Fig. 8E**). Staining for various subsets based on Ly49 and NKG family members and CD122 did not reveal major changes in mice treated with PI3K inhibitors (**Fig. 8F, G**). GDC-0941 caused a small but significant decrease in the percentage of Ly49A^+^ and Ly49C/I^+^ cells, and MLN1117 also decreased the Ly49C/I^+^ compartment. Overall, these data indicate that class I PI3K inhibition in mice for one week does not cause general lymphopenia or reduce NK cell numbers, nor does it strongly dysregulate NK homeostasis. Further experiments are necessary to evaluate the effects of different PI3K inhibitors on CMC, ADCC and cytokine production *in vivo*.

## Discussion

p110α is an important cancer drug target but is expressed ubiquitously [4], [25], [42]. Therefore, it is essential to define the function of this PI3K isoform in normal tissues and cells, particularly those that modulate tumor growth and survival. Here we used new chemical tools to probe the function of p110α in NK cells. We compared the effects of p110α inhibitors to the impact of other isoform-selective inhibitors, and to compounds with a pan-PI3K profile. The results show that pan-PI3K inhibition strongly suppresses CMC, ADCC and cytokine production by NK cells, whereas inhibition of p110α has much lesser impact on NK cell function *in vitro*. We used two different p110α inhibitors, MLN1117 and A66, at concentrations that suppress signaling and growth of *PIK3CA* mutant cancer cells [10], [11], [36]. Both compounds seemed to reduce CMC and release of certain cytokines, but in most cases these effects did not reach statistical significance. These findings suggest a potential advantage of p110α-selective inhibitors in clinical development for oncology for further study: relative to pan-PI3K inhibition, selective blockade of p110α should leave key NK cell functions intact such as ADCC. This property might prove particularly important in patients treated with PI3K inhibitors together with immunotherapeutic antibodies like trastuzumab.

Treatment of mice for one week with either GDC-0941 or MLN1117 had neglible effects on NK cell numbers or subset frequencies. These data fit with *in vitro* experiments showing lack of direct cytotoxic effects of PI3K inhibitors on NK cells (**Fig. S2**). It is possible that longer treatments *in vivo* would affect NK cell maturation or survival. It would be interesting to compare the effects of pan-PI3K and isoform-selective inhibitors on NK cell functions *in vivo*, such as lysis of tumor cells. We tried several models of NK-mediated rejection, including co-injection of differentially labeled RMA and RMA-S cells into the peritoneal cavity of syngeneic mice. Although we observed selective clearance of RMA-S cells, characteristic of an NK anti-tumor effect, we found that GDC-0941 treatment had no consistent effect compared to vehicle control (data not shown). A literature search did not identify any previous publications demonstrating that a chemical PI3K inhibitor impairs NK cell function *in vivo*. A likely explanation is that currently available pan-PI3K inhibitors are not tolerated at doses that completely inhibit PI3K activity, or that the pharmacokinetics are insufficient to maintain complete suppression of all PI3K isoforms in NK cells over the time course of tumor rejection models. The doses we used of GDC-0941 were sufficient to reduce marginal zone B cell populations, indicating inhibition of p110δ, but might not have inhibited other isoforms to the same extent. Further work is required to establish a system in which NK cell function is reduced by a pan-PI3K inhibitor, in order to compare with isoform-selective compounds.

Our data add to the knowledge base of p110γ and p110δ function in murine NK cells. The role of these isoforms has been investigated previously using mice with null or inactivating mutations in the *Pik3cg* and *Pik3cd* genes encoding these catalytic subunits [24], [27]–[30]. Genetic inactivation of p110γimpairs NK cell maturation, and NK cells lacking this isoform display various defects in response to chemokines and activatory receptors [24], [28], [29]. However, a previous study reported that acute inhibition of p110γwith AS252424 did not affect CMC or IFN-γ production by wild-type NK cells [24]. Our data confirm that AS252424 does not impede CMC or production of numerous cytokines by NK cells. It is possible that impaired function of NK cells with genetic p110γ deficiency results from development defects.

In contrast, there are consistent data that p110δ has a measureable contribution to NK cell biology. Earlier studies using p110δ kinase-dead knockin mice demonstrated a reduced NK cellularity and a selective impairment in the terminal maturation of Ly49C and Ly49I NK subsets and an alteration in CD27 and CD11b-based NK subsets [27]. Several studies have also shown reduced CMC and cytokine production by NK cells lacking p110δfunction [24], [27], [29], and others showed impaired chemotaxis and CMC using either genetic inactivation or treatment with IC87114 [28], [30]. Consistent with these reports, we found that IC87114 (1 µM) reduced mouse NK CMC towards RMA-S and YAC-1 cells and diminished production of IFN-γand some other cytokines. However, IC87114 did not significantly reduce human NK CMC towards K562 cells. Whether p110δinhibitors currently in clinical trials interfere with NK cell function in humans will be important to address. Thus far, the clinical data reported at conferences indicate favorable responses to the combination of CAL-101 and rituximab, suggesting that p110δ inhibition does not greatly interfere with the therapeutic mechanism of the antibody [4]. However, inhibiting p110δ might affect NK cell extravasation and tumor infiltration efficiency [28]. p110δinhibitors may also impair the function of cytotoxic T lymphocytes [43].

The p110βisoform of PI3K is unusual in that it can be activated downstream of both tyrosine kinases and G protein coupled receptors [13], [44], [45]. Inhibitors of p110βor dual p110α/β inhibitors have been considered as possible options in human tumors driven by PTEN loss [4]. Our data indicate that while p110β protein expression is barely detectable, inhibiting p110β has a modest effect on NK cells functions similar to inhibiting p110α. The dual p110α/β inhibitor INK1316 generally had greater effects on NK cell functions than the single inhibitors. Together with the results using IC87114 and the pan-PI3K inhibitors, these data support the conclusion that multiple class I PI3K isoforms (p110α, p110β and p110δ) all provide overlapping contributions to NK cell function in response to activating ligands or missing self.

The anti-cancer activity of compounds targeting PI3K enzymes will depend not only on direct effects on tumor cells but also on indirect modulation of immune cells. The immune effects of PI3K inhibitors will also influence the efficacy of immunotherapies used in combination. The data presented here suggest that selective p110α inhibition will preserve the function of NK cells, an important cell type in tumor immunosurveillance and immunotherapy.

## Materials and Methods

### Reagents

All reagents were purchased from Sigma-Aldrich (St. Louis, MO) unless otherwise noted. MLN1117 (MLN1117; p110α-selective inhibitor), A66 (p110α-selective inhibitor), and INK1316 (INK1316; dual p110α/β inhibitor) were obtained from Intellikine, Inc. (La Jolla, CA), now part of Millennium Pharmaceuticals. TGX-221 (p110β-selective inhibitor), AS-252424 (p110γ-selective inhibitor), IC87114 (p110δ-selective inhibitor), GDC-0941 (pan-class IA PI3K inhibitor), and ZSTK474 (pan-class IA PI3K inhibitor) were obtained from Chemdea (Ridgewood, NJ). AS-252424 was reconstituted in ethanol (EtOH) and the other inhibitors were reconstituted in DMSO. All aliquots were stored at −80°C and working solutions were prepared freshly, prior to the addition of inhibitors to cell cultures.

### Mice

All of the animal protocols used were approved by the Institutional Animal Care and Use Committee at UC Irvine. Female C57BL/6 mice six weeks of age were purchased from The Jackson Laboratory. On arrival, the mice were randomized, transferred to plastic cages containing sawdust bedding (5 mice per cage), and quarantined for 1 week. The mice were given food (Purina Certified Laboratory Chow) and water *ad libitum*. Animal holding rooms were kept at 21°C to 24°C and 40% to 60% relative humidity with a 12-h light/dark cycle.

### Cell lines

RMA, RMA-S, EL4, EL4^H60^ and YAC-1 cells were described previously [27]. CCRF-CEM, human acute T lymphocytic leukemia cell line was kindly provided by Dr. Matthew Janes (Intellikine, La Jolla, CA). The human NK cell line NKL was provided by Dr. Melissa Lodoen (UC Irvine). K562 cells were obtained from Dr. Tiong Ong (Duke-National University of Singapore). NK92 cells were from ATCC (Manassas, VA). All cells were cultured in RPMI1640 medium supplemented with 10% FBS, 100 U/ml penicillin-streptomycin, 2 mM L-glutamine, 5 mM HEPES buffer, and 50 mM 2- mercaptoethanol, which we refer to as complete RPMI. For NKL and NK92 cells, the media was supplemented with human IL-2 (500 U/ml).

### Human NK cell isolation

Peripheral blood mononuclear cells (PBMCs) were isolated from 25–30 ml of human buffy coat obtained from the donor center at the BloodCenter of Wisconsin, using Ficoll-Paque Plus (17-1440-02, GE Healthcare, Uppsala, Sweden). NK cells were isolated from 200–300 million PBMCs, using EasySep Negative Selection Human NK Cell Enrichment Kit (19055, Stemcell technologies, Vancouver, Canada).

### Cell purification and NK cell expansion

Spleens were aseptically isolated from the mice, and NK cells were purified by negative selection using the NK-cell isolation kit (Miltenyi Biotec, Auburn, CA). Negative magnetic selection was also used to purify T cells from lymph node, and B cells from spleens. Purified NK cells were expanded for 7–8 days in complete RPMI with 1000 U/ml recombinant human interleukin-2 (IL-2) (Frederick National Laboratory for Cancer Research, Frederick, MD). Purity of the NK cultures was determined by flow cytometry.

### Flow cytometry

Anti-mouse NK1.1 (PK136), CD3ε (145-2C11), IFN-γ (XMG1.2), NKG2D (A10), NKG2A/C/E (20d5), CD49b (DX5), CD122 (5H4), CD11b (M1/70), and CD27 (LG.7F9) were obtained from eBioscience. mAbs for Ly49A (A1), Ly49D (4E5), Ly49C/I (5E6), and Ly49G2 (4D11) were obtained from BD Biosciences. The antibodies were conjugated to fluorescein isothiocyanate, pacific blue, phycoerythrin, allophycocyanin, or peridinin chlorophyll protein. A standard flow cytometry analysis was performed using a FACS Calibur (BD Bioscience) with FlowJo software (TreeStar, Ashland, OR).

### Cytotoxicity assay

NK cell mediated cytotoxicity (CMC) and antibody-dependent cellular cytotoxicity (ADCC) were measured by calcein-AM release assay, with sensitivity similar to traditional ^51^Cr release assay (Roden et al., J Immunol Methods 1999; Neri et al., Clin Diagn Lab Immunol 2001; Tai et al., Cancer Res 2005). A total of 5×10^3^ target cells/well were mixed with NK effector cells at indicated E:T ratios in pentaplicates and incubated for 2–4 hours at 37°C in IL-2-free medium in round-bottomed 96-well plates. Target cells including RMA, RMA-S, EL4, EL4^H60^, YAC-1, CCRF-CEM and K562 cells were labeled with 10 µM calcein-AM (Molecular Probes, Eugene, OR), washed, and used for co-culture with NK cells. Indicated concentrations of PI3K inhibitors or AKT inhibitor were added to NK cells for 15 minutes at 37°C prior to co-culture with target cells. DMSO was used as vehicle control in the media and ethanol was used as vehicle control for AS252424. For ADCC, 2 µg/ml mouse anti-human CD4 mAb was added. After 2–4-hour co-culture, 100 µl culture supernatants were transferred to a 96-well black plate (Nunc) and arbitrary fluorescent units (AFU) were read on Molecular Devices Spectra MAX GeminiXS (485 nm excitation/538 nm emission). Calculation of percentage of specific lysis from pentaplicate experiments was done using the following equation: % specific lysis  = ((AFU mean experimental release - AFU mean spontaneous release)/(AFU mean maximal release - AFU mean spontaneous release)) x 100, where “AFU mean spontaneous release” is calcein release by target cells in the absence of NK cells and “AFU mean maximal release” is calcein release by target cells upon lysis by 2% Triton X-100. Cytotoxicity assays for NK92 or primary human NK cells were done with K562 cells as targets using a ^51^Cr release assay, as described [46].

### Cytokine and chemokine quantification

NK cells (5×10^4^ cells/well) were incubated for 15 min at 37°C in the absence or in the presence of PI3K inhibitors prior to stimulation. The cells were stimulated with 5 µg/ml of plate-bound anti-NKG2D (A10) or anti-NK1.1 (PK136) mAbs for indicated time periods. IFN-γ was measured by intracellular staining, ELISA, multiplexing assay, and RT-PCR. Other cytokines and chemokines were quantified by multiplexing assay. For intracellular staining, NK cells were stimulated with anti-NKG2D or anti-NK1.1 mAbs for 18 h in the presence of Brefeldin A for the last 4 h of activation. Where indicated in the figures, NK cells were activated with 10 ng/ml IL-12 and 1 ng/ml IL-18 in the presence of anti-NKG2D mAb (A10). Activated NK cells were Fc-blocked; stained for NK1.1 and CD3ε mAbs; fixed in 2% paraformaldehyde; permeabilized using 0.5% saponin; and quantified for intracellular IFN-γ using APC-conjugated anti-IFN-γ mAbs (eBioscience) through flow cytometry. The primary gate was drawn on the total population of viable cells and then gated based on surface NK1.1 and CD3ε expression. Intracellular IFN-γ levels were determined from the NK1.1^+^/CD3^−^ population. To determine the secreted cytokines and chemokines, NK cells were stimulated with anti-NKG2D mAbs in the absence or presence of PI3K inhibitors for 18 h and the supernatant was collected. IFN-γ was quantified by ELISA and multiplexing assay. Other cytokines and chemokines including IL-1α, IL-1β, IL-2, IL-3, IL-4, IL-5, IL-6, IL-9, IL-10, IL-13, IL-17, IL-12 (p40), IL-12 (p70), Eotaxin, G-CSF, GM-CSF, MCP-1, MIP-1α (CCL3), MIP-1β(CCL4), RANTES (CCL5), and TNF-α were quantified using Multiplex kit (Bio-Rad Laboratories). Sandwich ELISA for IFN-γ was performed with a Ready-set-go kit from eBioscience, and developed with TMB substrate for colorimetric detection after which the reaction was stopped with 1N sulfuric acid and read on a plate reader at 450 nm. For IFN-γ mRNA quantification, NK cells were activated for 6 h and harvested for RNA extraction using the TRI Reagent (Molecular Research Center, Cincinnati, OH). Total RNA was reverse transcribed into cDNA using iScript cDNA Synthesis Kits (BIO-RAD, USA). A real-time polymerase chain reaction (PCR) analysis was used to analyze the IFN-γ gene expressions according to the manufacturer's instructions. For IFN-γ, forward primer (5′-ATGAACGCTACACACTGCATC-3′) and reverse primer (5′-CCATCCTTTTGCCAGTTCCTC-3′) were used and L32 forward primer (5′-AAGCGAAACTGGCGGAAAC-3′) and reverse primer 5′-TAACCGATGTTGGGCATCAG-3′) were used as the reference gene. Real-time PCR was performed using the Bio-Rad iCycler iQ5 Detection System and the SYBR Supermix kit (Bio-Rad, Hercules, CA), with amplification for 40 cycles of denaturation at 95°C for 30 s, annealing at 53°C for 30 s, and extension at 72°C for 30 s. The threshold cycle number (Ct) was calculated with BIO-RAD software and relative transcript quantities were calculated using the 2^−ΔΔCt^ method with L32 as the reference gene which was amplified from the same samples. ΔCt is the difference in the threshold cycles of mRNA for selected genes relative to those of L32 mRNA and ΔΔCt is the difference in the ΔCt of inhibitor-treated group and vehicle-treated control group.

### Immunoblots

For measurement of AKT phosphorylation, whole-cell lysates (20 µg) were separated by 10% SDS-PAGE and electrotransferred to polyvinylidene difluoride (PVDF) membranes (Amersham Biosciences, Piscataway, NJ). The membranes were pre-incubated for 1 h at room temperature in Tris-buffered saline (pH 7.6) that contained 0.1% Tween-20 and 5% not-fat dry milk. The PVDF membranes were then incubated with specific antibodies against phosphorylated AKT (S473) or total AKT (Cell Signaling Technology). Total AKT was measured by stripping and reprobing. Immunoreactive bands were detected by incubating with horseradish peroxidase-conjugated anti-rabbit IgG (Cell Signaling Technology) and the bands were visualized using the ECL system (Amersham Biosciences). For measurement of p110 isoform expression, purified B, T and NK cells from C57BL/6 mice along with the SKOV and U87MG cell lines (ATCC, Manassas, VA) were lysed and 30 µg protein were separated by SDS-PAGE. Proteins were transferred to nitrocellulose and probed with the following antibodies to the different PI3K p110 isoforms: p110α (C73F8), p110β (C33D4), p110γ (D55D5) (Cell Signaling Technology), p110δ(Abcam, Cambridge, MA) and Actin (Sigma Aldrich).

### In vivo NK cell development

Wild-type C57BL/6 mice were orally dosed either with vehicle, MLN1117, or GDC-0941 using a sterile disposable 20G-1.5″ feeding needle (Fisher). Four mice per group were given the indicated drugs for 7 d before sacrificing on day 8. Spleen and bone marrow (BM) were isolated and analyzed for NK cell development by flow cytometry. In all cases, the vehicle group received both vehicles used to formulate the two different drugs.

### Statistical analysis

The means ± standard errors of the means (SEM) were determined for each treatment group in the individual experiments and/or three independent experiments. Statistical analysis was performed with the unpaired Student's *t* test or one-way ANOVA, with each condition compared to the stimulated-vehicle control. Each inhibitor except for AS-252424 was compared to DMSO vehicle; AS-252424 was compared to ethanol vehicle.

## Supporting Information

Figure S1 **Representative NK cell purity.** NK cells from C57BL/6 mice were purified from spleen and expanded for 7 days in IL-2. Purities of the NK cells from freshly isolated cells and 7 day-cultured cells were measured in CD3^−^NK1.1^+^ NK cells by surface staining and FACS.
(TIF)

Figure S2 **Pan-PI3K inhibition suppresses NK cell-mediated cytotoxicity.** NK cells from C57BL/6 mice were purified from spleen and expanded for 7–8 days in IL-2. Cytotoxicity was determined by calcein release assay with the NK cells and calcein AM-labeled target cells at the indicated effector:target (E:T) ratios. Percentage specific lysis was calculated from pentaplicate cultures as follows: ((mean experimental release - mean spontaneous release)/(mean maximum release - mean spontaneous release)) x 100. (**A**) Cytotoxicity was tested against RMA, RMA-S, EL4, or EL4^H60^ cells. NK cells were pre-treated with vehicle (0.1% DMSO) or 500 nM ZSTK474 for 15 min, followed by co-culture with calcein AM-labeled target cells for 4 h. (**B**) The dose-dependency of pan-PI3K inhibitor ZSTK474 was determined by calcein release assay with the NK cells and calcein AM-labeled RMA-S cells at 10∶1 E:T ratio in the presence of indicated concentrations of ZSTK474 for 3 h.
(TIF)

Figure S3 **Effects of PI3K inhibitors on cell viability.** Cell viability of effector and target cells was determined by trypan blue exclusion (upper panel) and calcein release assay (lower panels), respectively. NK cells from C57BL/6 mice were purified from spleen and expanded for 7–8 days in IL-2. NK cells were treated with vehicle (0.1% DMSO or 0.1% ethanol) or 1 µM indicated inhibitors (TGX-221 was 0.5 µM) for 4 h. The cells were collected and the cell viability was determined by trypan blue exclusion (upper panel). RMA-S and YAC-1 cells were labeled with calcein AM and treated with vehicle (0.1% DMSO or 0.1% ethanol) or 1 µM indicated inhibitors (TGX-221 was 0.5 µM) for 2 h. Culture supernatants were collected and calcein fluorescence was measured (lower panels). The data are expressed as the means ± SEM of three independent experiments. Statistical analysis was performed with one-way ANOVA using Prism 6 (GraphPad Software, Inc.) to compare the differences between vehicle and each inhibitor-treated group.
(TIF)

Figure S4 **Isoform-selective inhibitors have little effect on cytotoxicity of human NK92 cells.** K562 cells were labeled with ^51^Cr and co-cultured with human NK92 cells at the indicated E:T ratios in the presence of 1 µM indicated inhibitors (TGX-221, GDC-0941, and ZSTK474 were 0.5 µM) for 2 h. Specific ^51^Cr release was measured as in Figure 3A. The data are expressed as the average of two independent experiments.
(TIF)

Figure S5 **Representative FACS plots showing IFN-γ production in anti-NKG2D-stimulated NK cells.** The NK cells were stimulated with plate-bound anti-NKG2D mAb in the presence of 1 µM indicated inhibitors (TGX-221 was 0.5 µM). After 18 h stimulation, the NK cells were harvested and the intracellular IFN-γ level was determined by flow cytometry. Brefeldin A was added for the last 4 h before cell harvest and IFN-γ production was measured in CD3^−^NK1.1^+^ NK cells by intracellular staining. The results presented are representative of three independent experiments.
(TIF)
