# Supplementary figures and images for: Effects of Novel Isoform-Selective Phosphoinositide 3-Kinase Inhibitors on Natural Killer Cell Function
Source: PLoS One. 2014 Jun 10;9(6):e99486. doi: 10.1371/journal.pone.0099486 (PMC4051752; doi:10.1371/journal.pone.0099486)

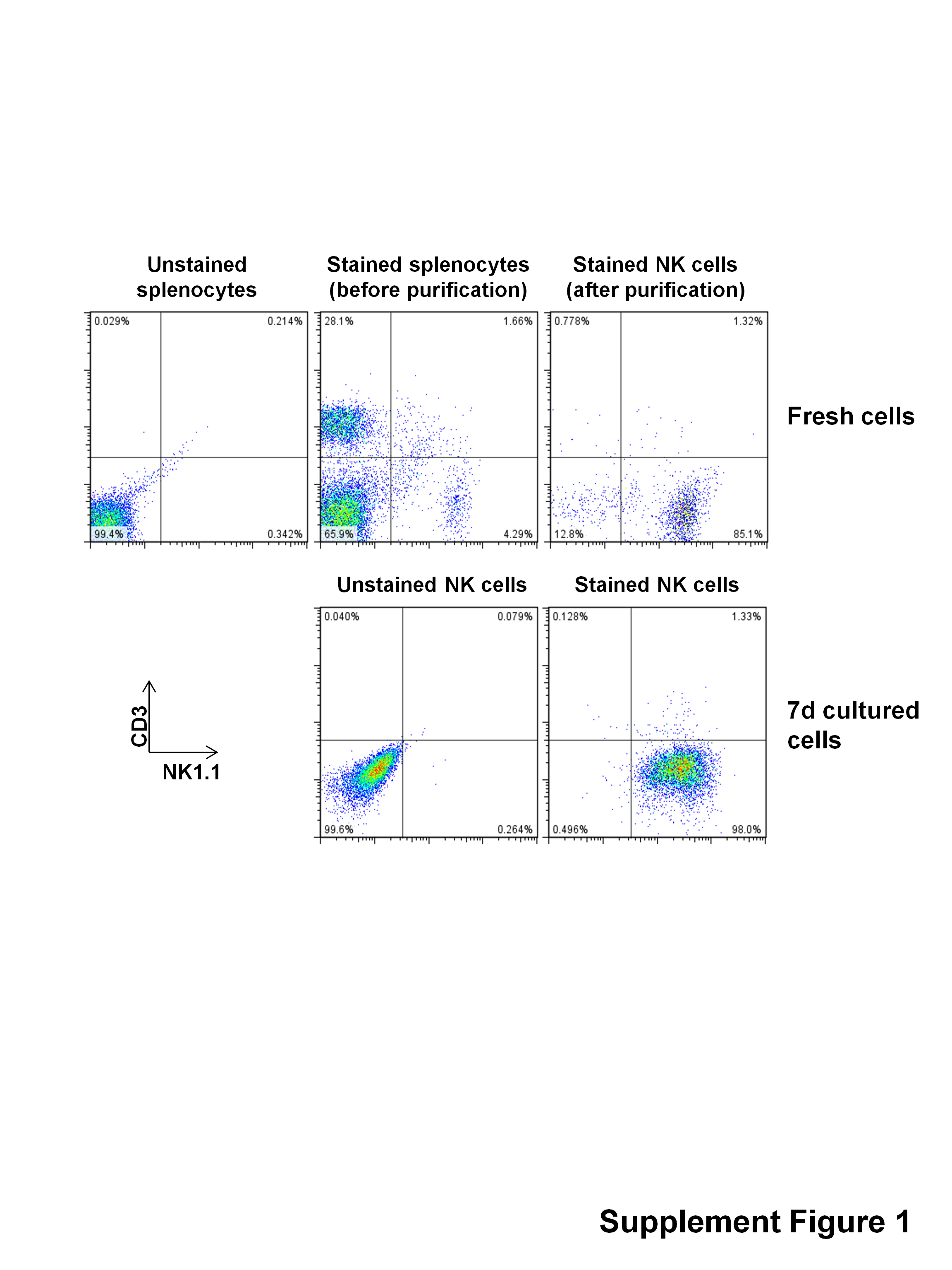

Supplement: Figure S1 — Representative NK cell purity. NK cells from C57BL/6 mice were purified from spleen and expanded for 7 days in IL-2. Purities of the NK cells from freshly isolated cells and 7 day-cultured cells were measured in CD3−NK1.1+ NK cells by surface staining and FACS. (TIF) [file pone.0099486.s001.tif]

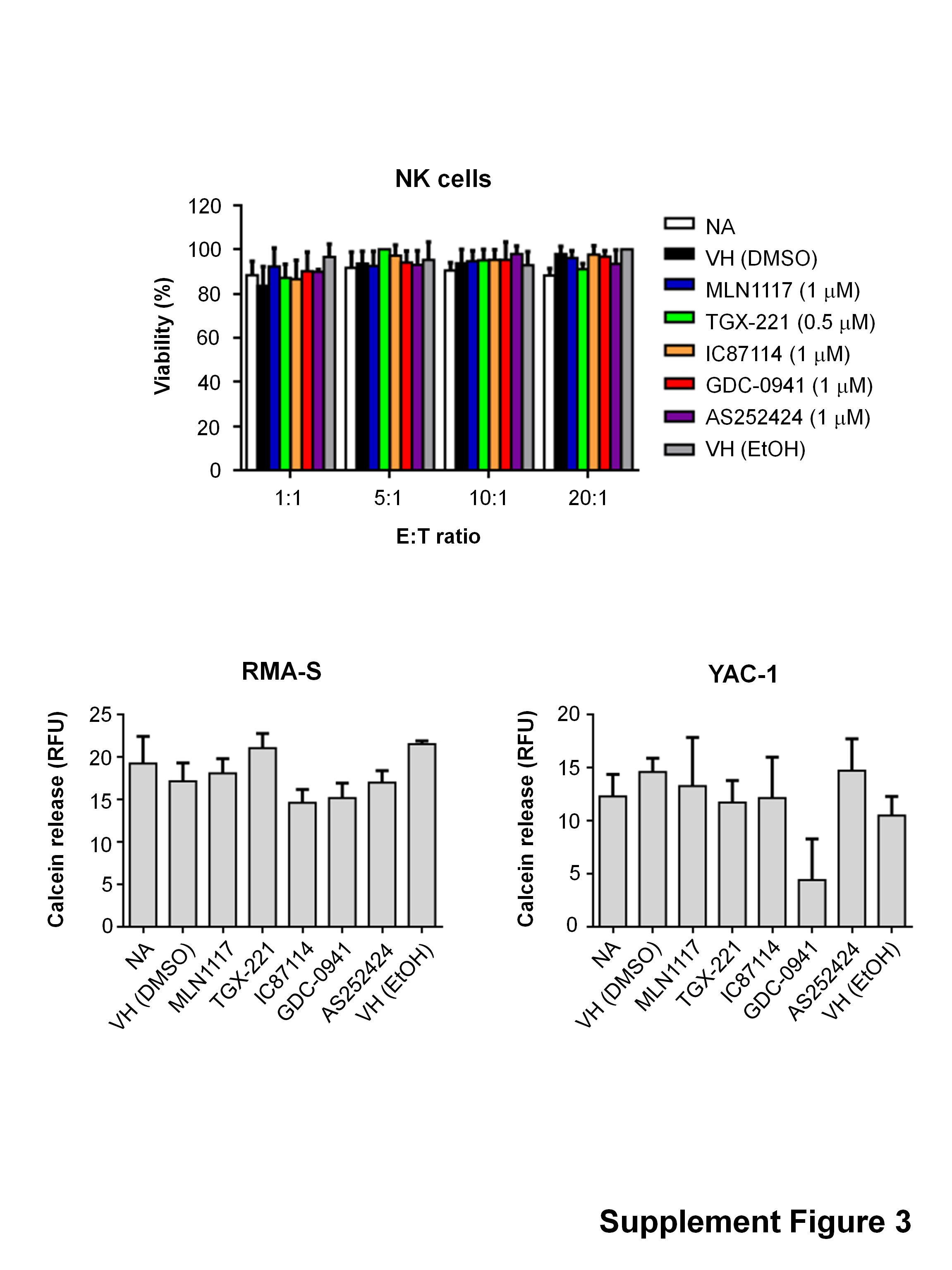

Supplement: Figure S3 — Effects of PI3K inhibitors on cell viability. Cell viability of effector and target cells was determined by trypan blue exclusion (upper panel) and calcein release assay (lower panels), respectively. NK cells from C57BL/6 mice were purified from spleen and expanded for 7–8 days in IL-2. NK cells were treated with vehicle (0.1% DMSO or 0.1% ethanol) or 1 µM indicated inhibitors (TGX-221 was 0.5 µM) for 4 h. The cells were collected and the cell viability was determined by trypan blue exclusion (upper panel). RMA-S and YAC-1 cells were labeled with calcein AM and treated with vehicle (0.1% DMSO or 0.1% ethanol) or 1 µM indicated inhibitors (TGX-221 was 0.5 µM) for 2 h. Culture supernatants were collected and calcein fluorescence was measured (lower panels). The data are expressed as the means ± SEM of three independent experiments. Statistical analysis was performed with one-way ANOVA using Prism 6 (GraphPad Software, Inc.) to compare the differences between vehicle and each inhibitor-treated group. (TIF) [file pone.0099486.s003.tif]

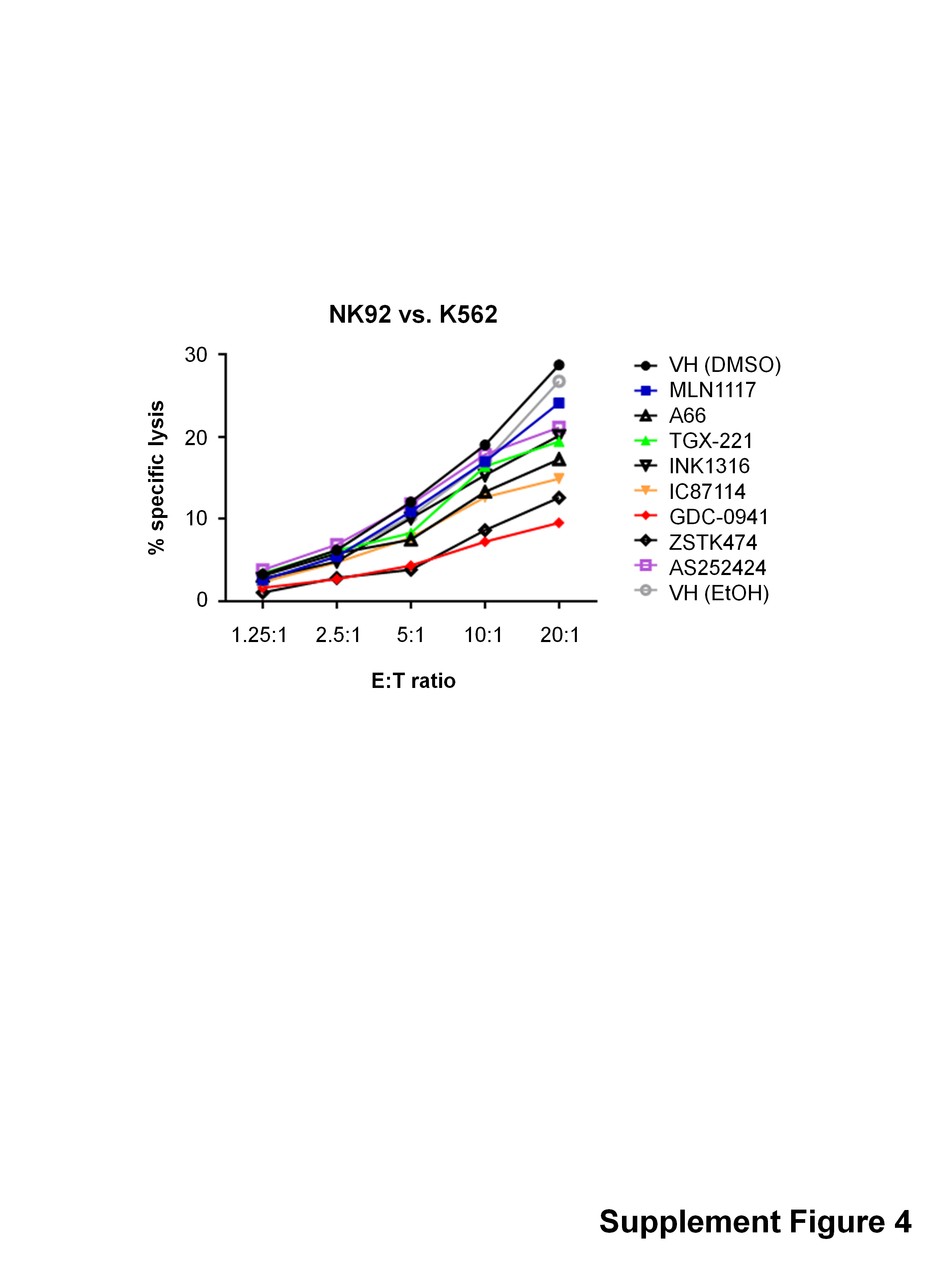

Supplement: Figure S4 — Isoform-selective inhibitors have little effect on cytotoxicity of human NK92 cells. K562 cells were labeled with 51Cr and co-cultured with human NK92 cells at the indicated E:T ratios in the presence of 1 µM indicated inhibitors (TGX-221, GDC-0941, and ZSTK474 were 0.5 µM) for 2 h. Specific 51Cr release was measured as in Figure 3A. The data are expressed as the average of two independent experiments. (TIF) [file pone.0099486.s004.tif]
